# EGYVIR: An immunomodulatory herbal extract with potent antiviral activity against SARS-CoV-2

**DOI:** 10.1371/journal.pone.0241739

**Published:** 2020-11-18

**Authors:** Wael H. Roshdy, Helmy A. Rashed, Ahmed Kandeil, Ahmed Mostafa, Yassmin Moatasim, Omnia Kutkat, Noura M. Abo Shama, Mokhtar R. Gomaa, Ibrahim H. El-Sayed, Nancy M. El Guindy, Amal Naguib, Ghazi Kayali, Mohamed A. Ali

**Affiliations:** 1 Central Public Health Laboratory, Ministry of Health and Population, Cairo, Egypt; 2 Center of Scientific Excellence for Influenza Viruses, National Research Centre, Giza, Egypt; 3 Biochemistry Department, Faculty of Science, Kafr El Sheikh University, Kafr El-Shaikh, Egypt; 4 Department of Epidemiology, Human Genetics, and Environmental Sciences, University of Texas, Houston, Texas, United States of America; 5 Human Link, Baabda, Lebanon; University of Hong Kong, HONG KONG

## Abstract

Due to the challenges for developing vaccines in devastating pandemic situations of severe acute respiratory syndrome coronavirus 2 (SARS-CoV-2), developing and screening of novel antiviral agents are peremptorily demanded. Herein, we developed EGYVIR as a potent immunomodulatory herbal extract with promising antiviral activity against SARS-CoV-2. It constitutes of a combination of black pepper extract with curcumin extract. The antiviral effect of EGYVIR extract is attributed to the two key phases of the disease in severe cases. First, the inhibition of the nuclear translocation of NF-kβ p50, attenuating the SARS-CoV-2 infection-associated cytokine storm. Additionally, the EGYVIR extract has an in vitro virucidal effect for SARS-CoV-2. The in vitro study of EGYVIR extract against SARS-CoV-2 on Huh-7 cell lines, revealed the potential role of NF-kβ/TNFα/IL-6 during the infection process. EGYVIR antagonizes the NF-kβ pathway *in-silico* and *in-vitro* studies. Consequently, it has the potential to hinder the release of IL-6 and TNFα, decreasing the production of essential cytokines storm elements.

## 1. Introduction

Over the last two decades, three novel zoonotic coronaviruses (CoVs) emerged to infect humans including the Severe Acute Respiratory Syndrome CoV (SARS-CoV) in 2002, the Middle East Respiratory Syndrome CoV (MERS-CoV) in 2012, and recently, the SARS-CoV-2 in late 2019 [1]. Although pathologies associated with viral infection with the three severe viruses are not yet completely understood, host-virus interaction plays a key role in the severity of disease as a result of triggering an immune response against the viral infection [2]. Excessive immune response due to viral infection is commonly associated with immune pathogenesis, inflammatory responses, and a cytokine storm which may result in poor outcomes such as acute respiratory disease syndrome (ARDS) and subsequently, multi-organ failure [3]. Modulating the immune response and decreasing the impact of the cytokine storm is important for defending against SARS-CoV-2 pathogenesis and improving outcomes.

Patients with SARS-CoV-2 infection mostly depend on their own immune defense to control the progress of infection, together with a non-specific treatment protocol to relieve symptoms and improve prognosis. To accelerate the application of specific medications to control SARS-CoV-2 infections, the Food and Drug Administration (FDA) approved drugs and plant-origin agents that represent ready-to-go materials for initial screening and safe use for COVID-19 patients.

Curcumin is the main active ingredient in the rhizome of turmeric (*Curcuma longa*). Curcumin has a variety of therapeutic properties including antioxidant, analgesic, anti-inflammatory, antiseptic activity, and anti-carcinogenic activity [4]. Curcumin is generally recognized as safe by the FDA. Curcumin was known to be safe for human consumption up to 12g/day during clinical trials without recording any side effects [5]. However, some studies indicated that high concentrations of curcumin effected directly on the genetic material in the nucleus as well mitochondrial DNA in cancer cell lines [6]. To overcome the drawbacks of bioavailability and rapid metabolism of curcumin, efforts were achieved to develop novel synthetic curcumin formulations [7].

Curcumin’s role as an antiviral agent has been previously reported against human immunodeficiency virus (HIV), Norovirus, Zika virus, Chikungunya virus, Herpes Simplex virus (HSV), Hepatitis viruses, and influenza type A viruses [8–11]. Curcumin targets critical steps of the virus replication cycle [12] and viral attachment/penetration [13]. Curcumin suppresses intercellular signaling cascades required for efficient virus replication [11]. Curcumin also plays an essential role in the attenuation of PI3K/Akt and NF-κB signaling pathways as well as targeting cellular transcription [14]. Piperine is a major active constituent of black pepper, known as an inhibitor of hepatic and intestinal glucuronidation and was also shown to improve the bioavailability of curcumin by 2000 times [15].

Purified extract of Curcumin and Piperine (PCP) is patent natural compounds that are constituted of a combination of black pepper extract with curcumin extract. Beside piperine and Curcumin, this formula is additionally containing multiactive ingredients including pentatricontane, sitosterol, termerone, lupeol, amyrines, and vitamin D3. Due to the challenges of rapidly developing vaccines during a pandemic, developing and screening of novel antiviral agents are urgently needed. In this study, we developed EGYVIR as a potent immunomodulatory herbal extract with a potent antiviral activity against SARS-CoV-2.

## 2. Materials and methods

### 2.1. Preparation of EGYVIR

*Curcuma longa* (Turmeric) root and *Piper nigrum* seeds were selected based on their ethno-medical importance. Healthy disease-free roots and seeds were purchased from local market in Egypt. The plant materials were dried and pulverized. A weight of 40 mg of well air-dried powder of *Curcuma longa* roots was infused in aqueous *Piper nigrum* seed extract (100ml) until complete exhaustion. The infusion was filtered through four-layered muslin cloth. Total concentration of obtained extract was 40 mg/L that was stored at 4°C till further use.

### 2.2. Gas Chromatography–Mass Spectrometry (GC-MS) analysis

To determine the chemical composition of the prepared EGYVIR extract, GC-MS was performed using the trace GC-TSQ Evo 8000 mass spectrometer (Thermo Scientific, Austin, TX, USA)with a direct capillary column TG–5MS (30 m x 0.25 mm x 0.25 μm film thickness). The temperature of the column oven was initially kept at 50 °C and then raised by 5 °C /min to 200 °C and kept for 2 min then raised to the final required temperature of 300 °C by 25 °C /min and kept for 2 min. The injector and mass spectrometry transfer line temperatures were held at 270 and 260 °C respectively. Helium gas was used as a carrier with a constant flow rate of 1 ml/min. Electron ionization (EI) mass spectra were collected at 70 eV ionization voltage over the range of m/z 50–650 in full scan mode. The components were identified by comparing their retention times and mass spectra with mass spectral libraries [16].

### 2.3. Cells and virus

Vero-E6 cells were cultured in Dulbecco’s modified Eagle’s medium (DMEM) (Lonza, Basel, Switzerland) containing fetal bovine serum (10%) (Lonza), and antibiotic antimycotic mixture (1%) (Lonza). The cells were incubated at 37 °C in a humidified atmosphere of 5% CO_2_. A SARS-COV-2, hCoV-19/Egypt/NRC-03/2020 (Accession Number on GSAID: EPI_ISL_430820) virus was propagated in VERO-E6 cells. The virus was titrated using plaque titration assay.

### 2.4. Cytotoxicity

To evaluate the *in vitro* cell viability of the prepared EGYVIR extract, the 3-(4, 5-dimethylthiazol -2-yl)-2, 5-diphenyltetrazolium bromide (MTT) assay was performed as previously described [17] with minor modifications. Briefly, cells were seeded in 96-well plates in DMEM supplemented with 10% fetal bovine serum, and 1% antibiotic antimycotic mixture. After 24 h of cell preparation, the growth medium was aspirated from each well and the cells washed with 1X phosphate buffered saline (PBS). Different concentrations of EGYVIR aqueous extract starting from 0.4 μg/ml were two fold serially diluted in DMEM then added to cultured cells in 96-well plate in triplicate and incubated for 24 h post treatment to determine the cytotoxic concentration 50 (CC50). The medium was then removed and the monolayer of cells washed with 1X PBS three times before adding MTT solution (20 μL/well of 5 mg/ml stock solution) and incubated at 37 °C for 4 h till formulation of formazan crystals. Crystals were dissolved using a volume of 200 μL of dimethyl sulfoxide (DMSO) and the absorbance measured at λmax 540 nm using an ELISA microplate reader. Finally, the percentage of cytotoxicity compared to the untreated cells was determined. The CC50 of EGYVIR extract was determined from a linear exponential equation.

### 2.5. Plaque reduction assay

The antiviral activity of EGYVIR extract was determined by plaque reduction assay [18] with minor modifications. Briefly, Vero-E6 cells were seeded in 6-well culture plates (10^5^ cells/ml) and incubated overnight at 37 °C under 5% CO_2_ condition. Previously titrated SARS-CoV-2 was diluted to optimal virus dilution, which gave countable plaques, and mixed with the safe concentrations of EGYVIR extract (0.4, 0.2, 0.1, 0.05 μg). The mixtures of virus and EGYVIR were incubated for 1 h at room temperature. Growth medium was removed from the 6-well cell culture plates and virus-extract mixtures inoculated in duplicate. After 1 h contact time for virus adsorption, 3 ml of DMEM supplemented with 2% agarose, 1% antibiotic antimycotic mixture, and 4% bovine serum albumin (BSA) (Sigma, St. Louis, Missouri, USA) were added to the cell monolayer then the plates were incubated at 37 °C for 3 days. The cells were fixed using 10% formalin solution for 1 h and the over layer was removed from each fixed well. Fixed cells were stained using 0.1% crystal violet in distilled water. Untreated virus was included in each plate as a control. Finally, plaques were counted and the percentage reduction in virus count recorded as follows:

Viralinhibition(%)=viralcountofuntreatedcells−viralcountofthetreatedcells/viralcountofuntreatedcellsx100

### 2.6. Time course analysis

The Vero-E6 cells (80–90% confluency) were infected at MOI of 0.1 of the virus then treated with EGYVIR and hydroxychloroquine as control at concentration 0.4 μg/ml and 2.2 μM, respectively. The cells were incubated in infection medium for 24, 48, and 72 h post infection at 37 °C in 5% CO_2_. Mock-infected cells without treatment were used as control. Cell culture supernatants were collected at each time point and virus was quantified by RT-qPCR.

Nucleic acid extraction for the cell culture isolates were done using the chemagic^™^ 360 instrument (Perkin Elmer, Waltham, Massachusetts, USA). Detection of SARS-CoV-2 RNA (ORF1 ab) was performed using Viasure Sars-CoV-2 Real Time PCR Detection Kit (CerTest Biotec, Zaragoza, Spain), the RT-PCR runs were done in triplicate and according to manufacturer’s recommendations. The obtained Ct values were changed to viral RNA copy numbers using a standard curve of ORF 1 ab assay, the viral inhibition in RNA copy number at each concentration was determined.

### 2.7. Mode of action

The mode of action of the tested extract was determined using three mechanisms, viral replication mechanism [19], viral adsorption mechanism [20], and virucidal mechanism [21]. The percentage of virus reduction of each mode of action was individually calculated based on untreated virus control wells.

### 2.8. TNF-α, IL-6, NF-κB p50 and total Ikβα levels of treated and untreated Huh 7 cells with EGYVIR post infection measured by enzyme-linked immunosorbent assay

The human hepatocellular carcinoma (Huh7) cells were plated at 1.8×10^5^ cells/well in 6-well plates and incubated overnight in DMEM. Cells were then infected with SARS-CoV-2 virus at MOI 0.1 with and without EGYVIR extract at concentration of 0.4 μg/ml in triplicate. Supernatants were collected at 0, 2, 8, 16, and 24 h post infection and centrifuged. TNF-α, IL-6 and total Ikβα concentrations were assayed using ELISA (R&D, Minneapolis, Minnesota, USA) according to the manufacturer’s recommendations. For evaluation of cytoplasmic and nuclear NF-κB p50 levels, cell supernatants were collected at the above time point then nuclear and cytoplasmic extraction were done using NE-PER nuclear and cytoplasmic extraction reagents (Thermo scientific, Waltham, Massachusetts, USA) according to the manufacturer’s recommendations. Then NF-κB p50 ELISA was done using NF-κB p50 colorimetric transcription factor assay kit (Abcam, Cambridge, UK).

### 2.9. TNF-α, IL-6, NF-κB p50 and total Ikβα levels of treated and untreated Huh 7 cells with EGYVIR post infection measured by reverse transcription-polymerase chain reaction

Huh7 cells were plated at 1.8×10^5^ cells/well in 6-well plates and incubated overnight in DMEM. Cells were then infected with SARS-COV-2 virus at MOI 0.1 alone or with EGYVIR extract 0.4 μg/ml in triplicate and cell supernatants were collected at 0, 2, 4, 6, 8, 10, 12, 16, 24 and 36 h post infection and centrifuged. The total RNA was then extracted from cells using Qiagen extraction kit according to the manufacturer’s protocol (Qiagen, Hilden, Germany). Subsequently, 500 ng of the purified RNA were used to synthesize the complementary DNA (cDNA) with random hexamer primers (Thermo Scientific) and Revert Aid H Minus M-MuL V Reverse Transcriptase (Thermo Scientific) according to the manufacturer’s protocol. The quantitative real-time PCR (qRT-PCR) reaction mixture (25 μl) comprises the following: 0.5 μl of cDNA template, 12.5 μl of Maxima SYBR green PCR master mix (Thermo Scientific) and 1 μl of each primer (100 μM forward and reverse primers). Reactions were run in triplicate on Applied Biosystems 7500 real-time PCR system (Applied Biosystems, Foster City, California, USA). The cycling conditions were as follows: 2 min at 50 °C, 2 min at 95 °C, cDNA were amplified by 45 cycles of PCR, with each cycle consisting of 30 s at 94 °C, 30 s at 52 °C, and 30 s at 72 °C. The primer sequences were as follows: for TNFα, forward,5‘-CCCAGGCAGTCAGATCATCTTC-3‘, reverse,5‘-GCTGCCCCTCAGCTTGA-3‘ [NM_000594.2]; IL-6, forward, 5‘-TACCCCCAGGAGAAGATTCC-3, reverse, 5‘-TTTCAGCCATCTTTGGAAGG-3‘ [NM_000600.3]; Ikβα Forward primer (5-CAGCAGACTCCACTCCACTT-3) Ikβα Reverse primer (5-GAGAGGGGTATTTCCTCGAA-3) and for β actin, forward (5- CACCATTGGCAATGAGCGGTTC -3) and reverse (5-AGGTCTTTGCGGATGTCCACGT -3). (NM_001101). Ct values were normalized to the values of the control β-actin house-keeping transcripts and log fold change was calculated according to the equation of 2^-ΔΔct [22].

### 2.10. Molecular docking

The Crystal structure of SARS-CoV-2 spike protein and P50 protein were obtained from protein database under numbers 6LZG and 1VKX, respectively. Ligand structures of ingredients of EGYVIR were obtained from zinc AC and chemspider databases and were converted to MOL2 format and adjusted for docking by UCSF Chimera as required prior to submission. The potential binding sites of each of the ligands to the target were determined using EA Docking provided by SwissDock using default parameters. After submitting each ligand and target, protein-ligand binding energy was scored using the CHARMM22 force field. Favorable clusters of lowest energy poses were visualized, manipulated, and analyzed by UCSF Chimera. Calculations were performed using switch dock server that evaluates protein-ligand binding energy using a scoring function based on the CHARMM22 force field.

### 2.11. Statistical analysis

Data were summarized by means ± SD of triplicates and compared by one-way ANOVA with post hoc Fisher’s least significant difference test. P-value < 0.05 was considered significant.

## 3. Results

### 3.1. GC-MS analysis of the EGYVIR

**Fig 1:**
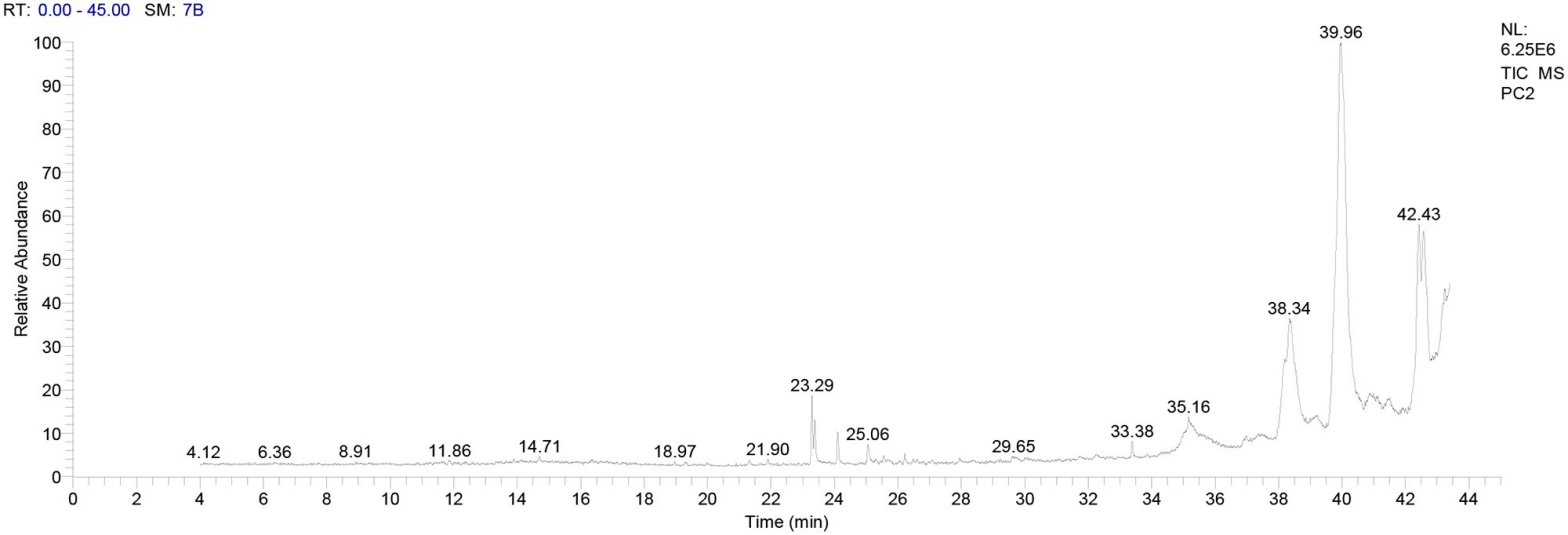
Chromatograms of GC-MS analysis of EGYVIR extract.

The prepared EGYVIR was analyzed using GC-MS to determine the amounts of the main components present. The active principles with their retention time (RT), molecular formula, molecular weight (MW), peak area in percentage are presented in Table 1. The EGYVIR extract included fifty- three ingredients. The major ingredients are Pentatricontane (41.04%), Amyrin (9.49%), Lupeol (8.86%), Turmerone (8.13%), Sitosterol (7.61%), Bisdemethoxycurcumin (6.8%), Piperine (4,6%), Vitamin D3 (1.76%), and Curcumin (1.3%) (Fig 1).

**Table 1:** Phyto-constituents present in EGYVIR extract as identified by GC-MS analysis.

Retention time	Compound Name	Area %	Molecular Formula	Molecular Weight	Cas #	Library
14.08	Piperine	4.6%	C_17_H_19_NO_3_	285.3	94-62-2	WileyRegistry8e
23.29	Turmerone	8.13%	C_15_H_22_O	218	82508-14-3	WileyRegistry8e
25.06	Bisdemethoxycurcumin	6.8%	C_19_H_16_O_4_	308.3	24939-16-0	WileyRegistry8e
38.35	á-Sitosterol	7.61%	C_29_H_50_O	414	83-46-5	replib
39.96	pentatricontane	41.04%	C_35_H_72_	492	630-07-9	WileyRegistry8e
42.42	α-Amyrin	9.49%	C_30_H_50_O	426	638-95-9	mainlib
42.58	Lupeol	8.86%	C_30_H_50_O	426	545-47-1	replib
23.29	1,25-Dihydroxyvitamin D3, TMS derivative	1.76%	C_30_H_52_O_3_Si	488	55759-94-9	mainlib

### 3.2. Cytotoxicity of the EGYVIR extract

**Fig 2:**
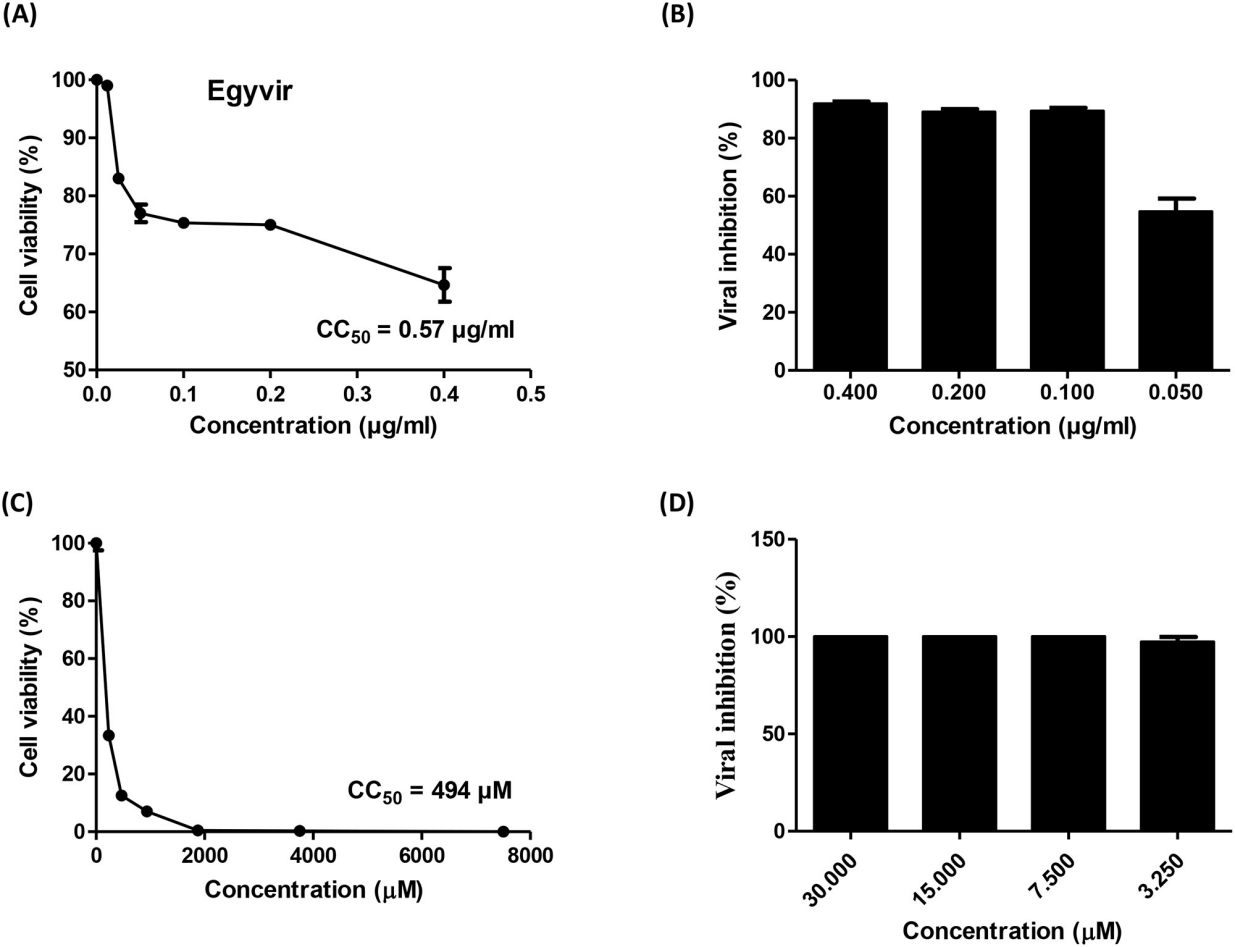
EGYVIR extract cytotoxicity and viral inhibition activity on Vero-E6 cell lines. (A and B) The cytotoxicity and antiviral activity of EGYVIR on SARS-COV-2 based on the dose response (0.4 to 0.05 μg/ml) was determined using MTT and plaque reduction assay, respectively. The result showed that the 50% cytotoxic concentration (CC50) was 0.57 μg/ml. The concentrations from 0.4 to 0.1 μg/ml showed 92% to 88% SARS -CoV-2 inhibition. At 0.05 μg/ml of EGYVIR, the viral inhibition decreased to 54.6%. (C and D) the cytotoxicity and antiviral activity of hydroxychloroquine as a reference drug, respectively.

The cytotoxicity of the EGYVIR extract was evaluated in Vero-E6 cells using MTT assay. The EGYVIR was almost not toxic for Vero-E6 cells up to a dose of 0.57 μg/ml (Fig 2A). The toxic effect of tested extract was dose-dependent. The result showed that the cytotoxic concentration 50 (CC50) value of EGYVIR was 0.57 μg. Therefore, for further studies we selected the safe concentrations of 0.05–0.4 μg/mL for subsequent antiviral studies.

### 3.3. Antiviral efficacy of EGYVIR extract

The antiviral activity of EGYVIR against SARS-CoV-2 was determined using plaque reduction assay. The result showed that the 50% inhibitory concentration (IC50) of EGYVIR was 0. 57 μg/ml (Fig 2B). The concentrations from 0.4 to 0.1 μg/ml showed 92% to 88% SARS-CoV-2 inhibition. At 0.05 μg/mL of EGYVIR, the viral inhibition decreased to 54.6% (Fig 2B and 2C).

### 3.4. Time course analysis

**Fig 3:**
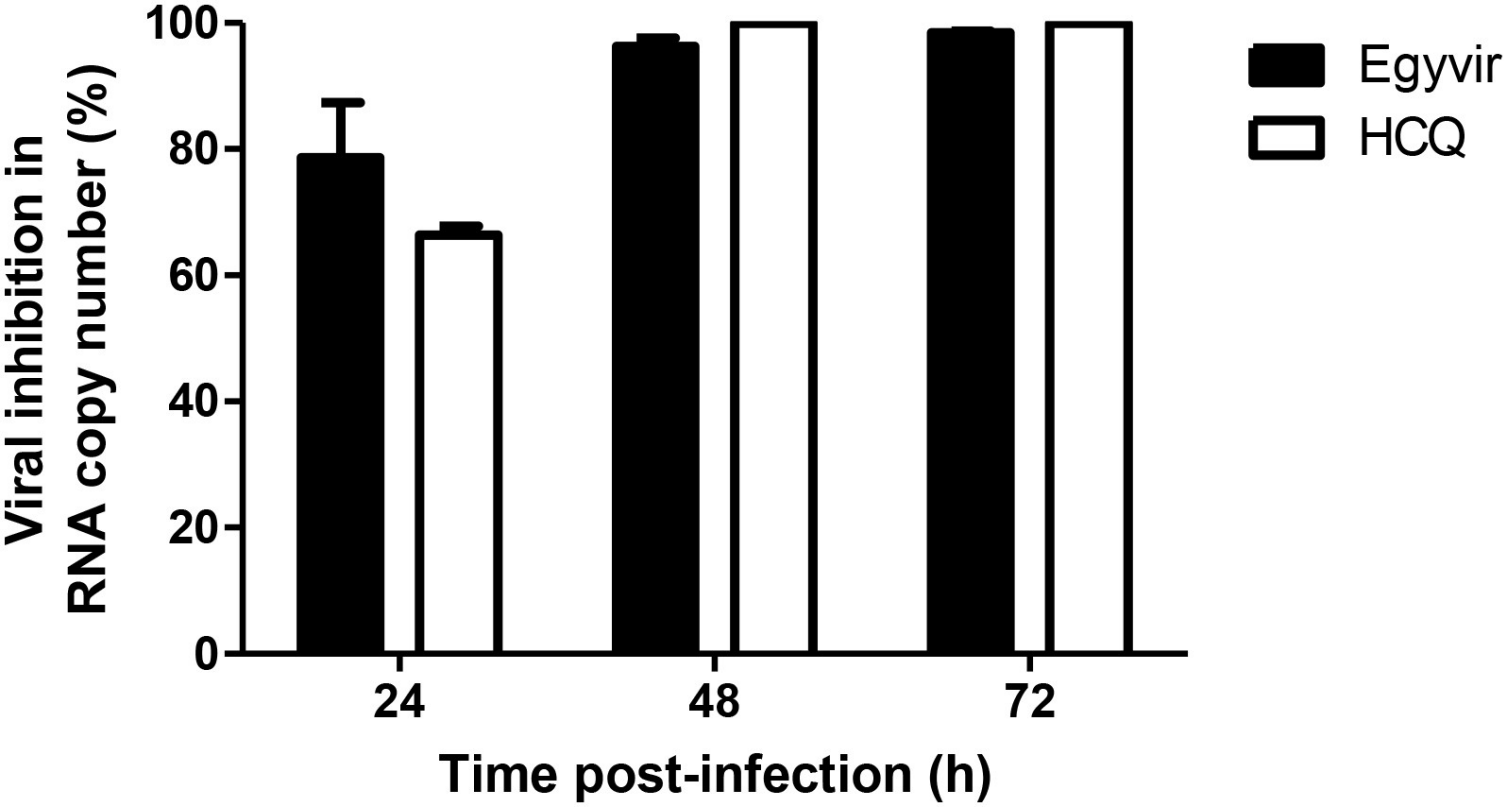
The inhibitory effect of EGYVIR extract (Extract) on Vero cells vs hydroxychloroquine (HCQ).

The infected Vero-E6 cells with SARS-CoV-2 virus in the presence of EGVIR showed a decrease in viral titer (78%) assessed by RT-qPCR (ORF1ab) after 24 h of infection. Also, the treated cells with hydroxychloroquine showed decreasing in viral inhibition (66%) compared to untreated cells. At 48 and 72 h of infection, a significant decrease of viral replication in the infected cells in the presence of either EGYVIR or hydroxychloroquine was noted in comparison to cells infected without treatment (Fig 3).

### 3.5. Antiviral mechanism of EGYVIR action

To understand the main action mechanism of the effective EGYVIR extract against SARS-CoV- 2 virus, we considered three main possible antiviral mechanisms: (i) inhibited attachment of virus to infected cell membrane, blocking the viral entry (viral adsorption); (ii) direct action to destroy or deformation of the virus surface proteins (virucidal activity); and (iii) inhibition of viral replication. The above-mentioned mechanisms could account for antiviral activities either independently or in combination. In addition, the viral-infection stage plays an important role in targeting viruses.

**Fig 4:**
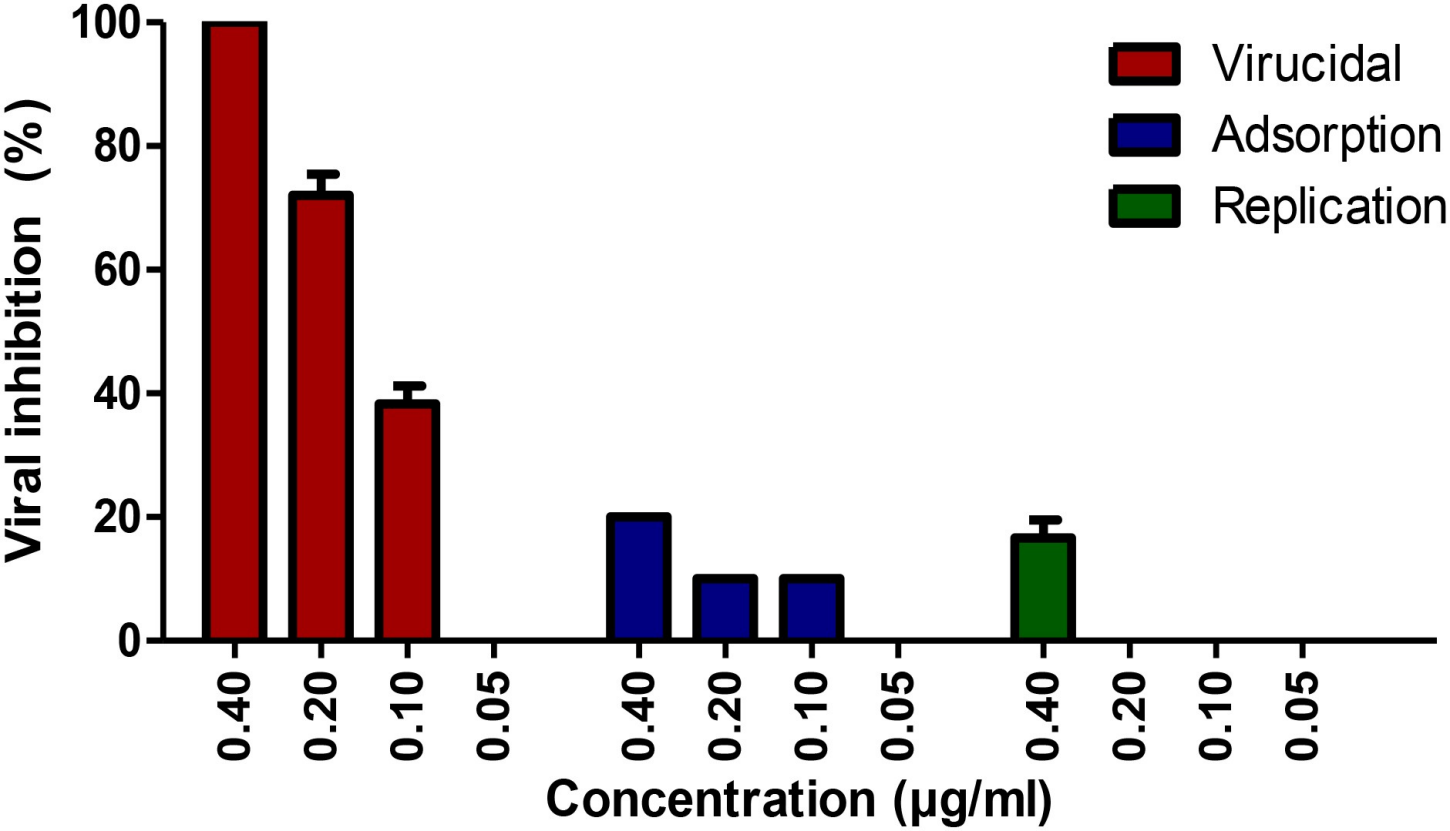
Mode of action of EGYVIR on Vero E6 cells.

EGYVIR showed virucidal effects on SARS-CoV-2 as showed in Fig 4. A negligible reduction in plaque formation was characterized in case of viral adsorption as well as virus replication mechanism.

### 3.6. Down regulation of Ikβα, TNF-α and IL-6 released from Huh7cells by treatment with EGYVIR extract

To investigate whether SARS-COV-2 virus can promote cytokine expression in Huh7 cells, we first measured the level of Ikβα, TNF-α and IL-6 in the supernatant of infected Huh7 cells. The levels of Ikβα, TNF-α and IL-6 in the culture supernatant of Huh7 cells were measured using enzyme-linked immunosorbent assay kits. SARS-CoV-2 virus induced the production of Ikβα, TNF-α and IL-6 in the culture supernatant of Huh7 cells in a dose and time dependent manner.

**Fig 5:**
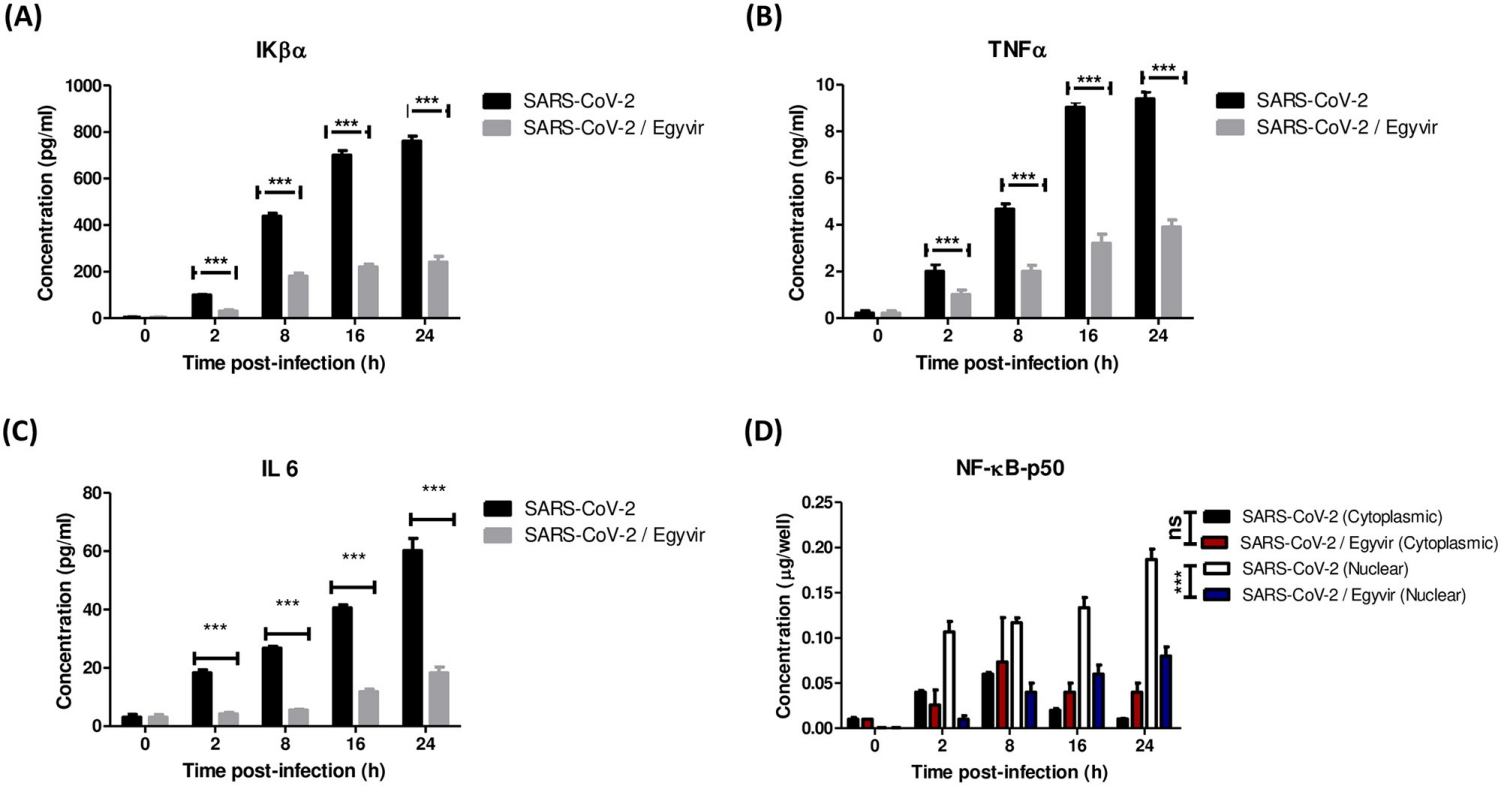
Protein levels of pro-inflammatory cytokines IL-6, TNFα, and NF-κB. (A) HUH7 cells treated with EGYVIR significantly down regulates IκBα levels at studied time point compared with SARS-COV-2 infected cells. (B) HUH7 cells treated with EGYVIR significantly down regulates TNFα levels at studied time point compared with SARS-COV-2 infected cells. (C) HUH7 cells treated with EGYVIR significantly down regulates IL-6 levels at studied time point compared with SARS-COV-2 infected cells. (D) EGYVIR significantly attenuates the nuclear translocation of p50 subunit in HUH7 cells compared with the SARS-COV-2 infected cells where the nuclear translocation became obvious after 2h post infection and significantly stable for 24h post infection.

Then we repeated the experiment with the infected Huh7 cells with the SARS-COV-2 virus in the presence and absence of EGYVIR extract. The supernatant of infected cells in two cases were collected at a time ranging from 0–24 h and the levels of IL-6, TNF-α, and Ikβα Huh7 were measured using ELISA. EGYVIR extract were significantly (p<0.001) down regulating the levels of IL-6, TNF-α and Ikβα in the culture supernatant of infected Huh7 cells at interval time points (Fig 5).

**Fig 6:**
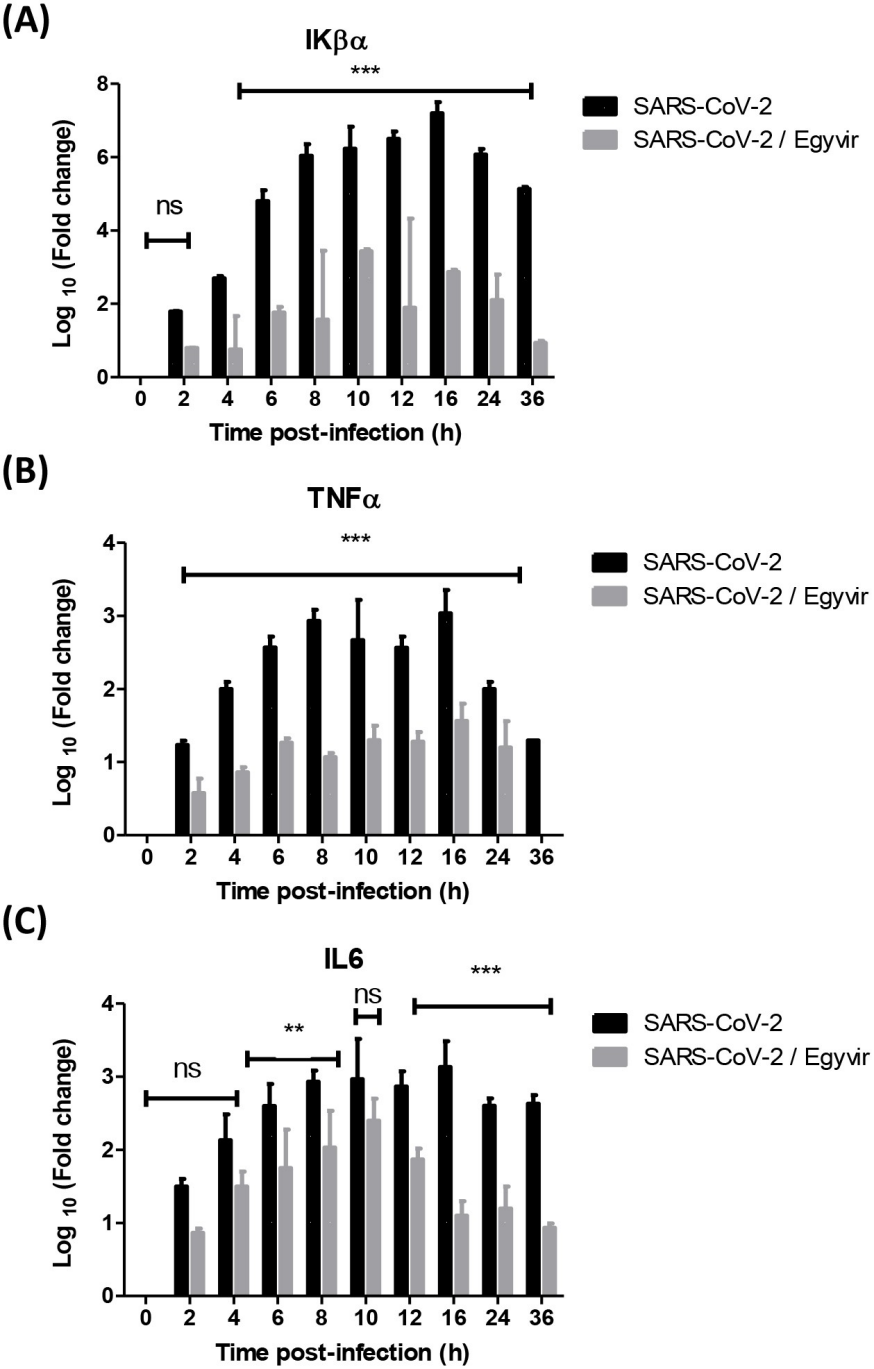
Transcriptional levels of pro-inflammatory cytokines Iκβα, TNFα, and IL-6. (A) EGYVIR down regulates mRNA levels of total Ikβα that affected the proteosomal degradation of the Ikβα that attenuates the NF-κB nuclear translocation. (B) EGYVIR down regulates the log fold change of TNFα induced by SARS- COV-2 infection by 2–3 times at the studied time points. (C) EGYVIR down regulates the log fold change of TNFα induced by SARS-COV-2 infection by 2–3 times at the studied time points.

To further determine if IL-6, TNF-α, and Ikβα levels occurred at the transcriptional level, IL-6 and TNF-α mRNA levels were evaluated by RT-PCR and normalized to β actin and log fold change was calculated. Huh7 cells were infected with SARS-CoV-2 at MOI 0.1 with and without EGYVIR extract at concentration of 0.4 μg/mL. Data showed that SARS-COV-2 strongly induced the transcription level of IL-6, TNF-α, and Ikβα, which was consistent with the release of IL-6, TNF-α, and Ikβα in the supernatants of infected cells. The treated cells with EGYVIR extract were significantly down regulating the transcriptional levels of TNF-α and Ikβα by 2–4 folds at all time points (Fig 6A and 6B). The IL-6 was significantly down regulated in the treated cells compared to untreated cells after infection with SARS-CoV-2 at 6–8 h (p<0.05), and 12–36 h (p<0.001). No significant difference (p>0.05) in the level of IL6 was recorded at 0-4h and at 10 h post infection (Fig 6C).

### 3.7. Effective EGYVIR extract action in blocking nuclear translocation of NF-κB p50

To monitor the activation status of NF-κB p50, we used enzyme-linked immunosorbent assay kits in triplicate independent experiments, the samples were collected at a time point range between 0–24 h and Huh7 cells supernatant collected at each time point and then cytoplasmic and nuclear extraction were done and the levels of nuclear and cytoplasmic NF-κB p50 were detected in three independent experiments. EGYVIR significantly attenuated the nuclear translocation of p50 subunit in Huh7 cells compared with the SARS-COV-2 infected cells where the nuclear translocation became obvious after 2 h post infection and significantly stable for 24 h post infection (Fig 5D).

### 3.8. Molecular docking

**Fig 7:**
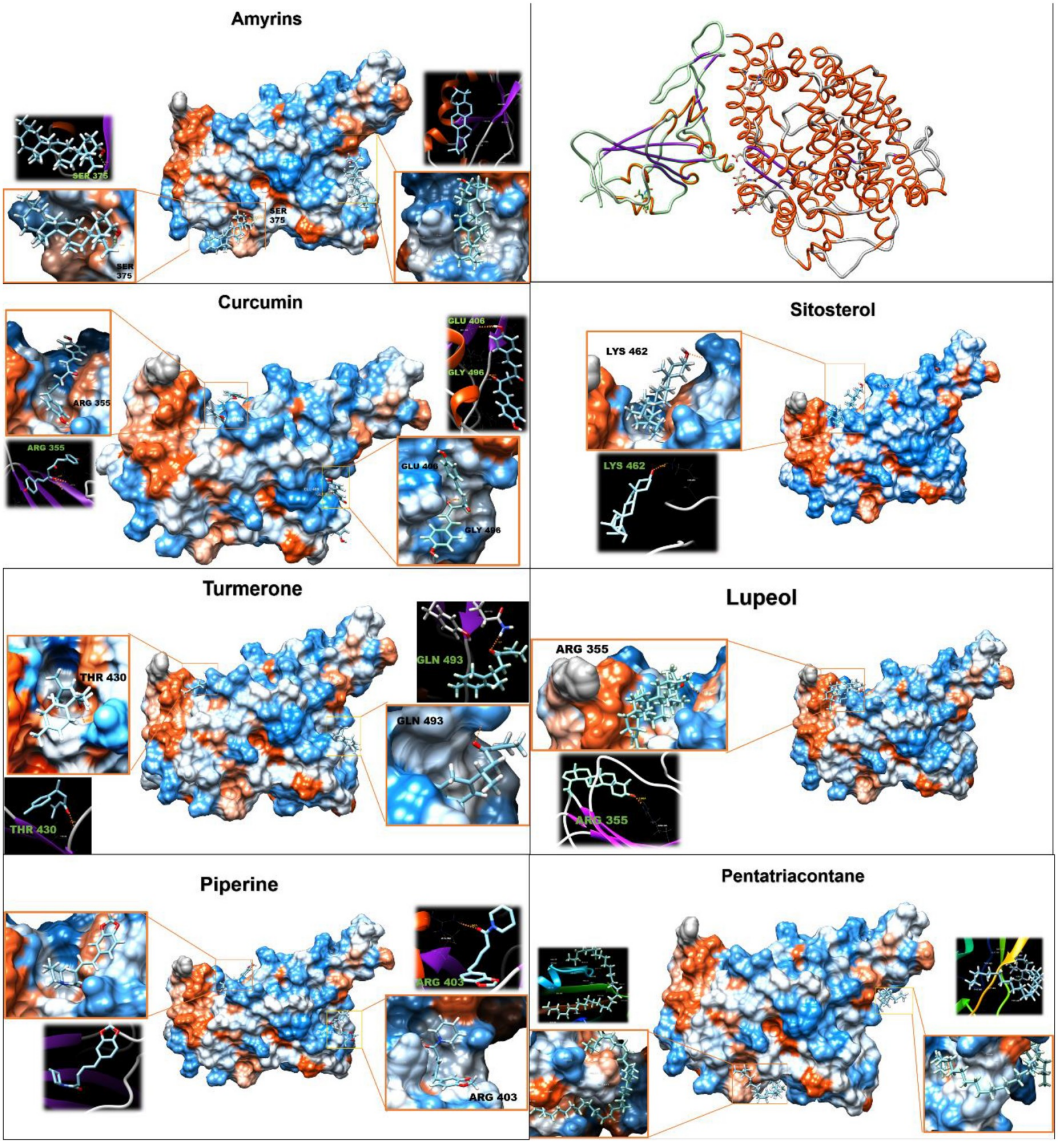
Molecular docking of EGYVIR ingredients on RBD of SARS-COV-2.

**Fig 8:**
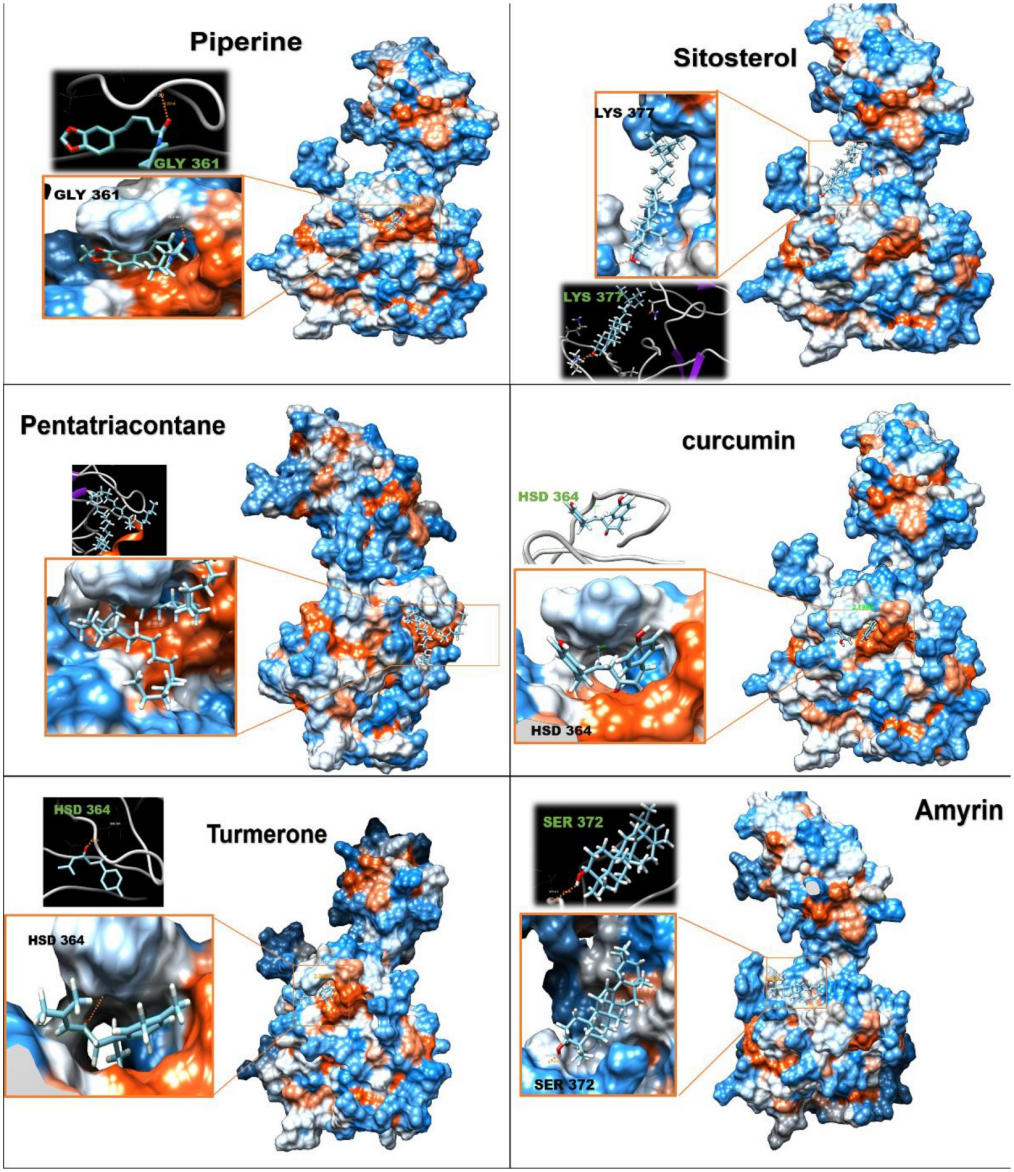
Molecular docking of EGYVIR ingredients on NFkB p50 subunit.

Based on the mode of action of EGYVIR on SARS-COV-2, docking analysis of the main ingredients of EGYVIR (ligands) with spike RBD and with P50 proteins were performed (Figs 7 and 8). The rationale behind the choice of RBD was that the drug had a direct effect on the virus itself (virucidal). Interaction energy calculations suggest that all components of EGYVIR (except sitosterol and lupeol) bind directly to the spike protein at the site of its interface with ACE2 receptor as shown in the structure of novel coronavirus spike receptor-binding domain complexes with its receptor ACE2 (PDB code: 6LZG). Energy calculations also showed that Pentatriacontane, curcumin, piperine, sitosterol, turmeron, lupeol and alpha-amyrines are predicted to bind with the RBD of spike protein in places other than the interface with estimated delta G ranging from -6 to -9.7 kcal/mol. Regarding the docking of EGYVIR components with P50, all components were found to bind with it, except lupeol. All delta G results were lower than -7 (Table 2).

**Table 2:** Showed chemical structure and molecular docking of ingredients of EGYVIR with viral spike RBD, and cellular P50 subunit.

				Spike RBD ACE2 interface			Spike RBD			p50		
Accession number	Ligand name	Chemical formula	Molar mass	FullFitness (kcal/mol)	Estimated ΔG (kcal/mol)	H Bond	FullFitness (kcal/mol)	Estimated ΔG (kcal/mol)	H Bond	FullFitness (kcal/mol)	Estimated ΔG (kcal/mol)	H Bond
ChemSpider ID11907	Pentatriacontane	C_35_H_72_	492.96	-844.0	-8.2	-	-861.1	-9.7	-	-2310.9	-9.6	-
ChemSpider ID4474770	Curcumin	C_19_H_16_O_4_	308.328	-794.8	-7.0	GLU 406, GLY496	-791.8	-7.3	ARG 355	-2223.9	-8.6	HSD 364
ZINC1529772	Piperine	C_17_H_19_NO_3_	285.34	-800.8	-6.4	ARG 403	-802.6	-7.2	-	-2260.8	-8.1	GLY 361
ZINC4095717	Sitosterol	C_29_H_50_O	414.71	0.0	0.0	-	-755.6	-7.2	LYS 462	-2211.0	-7.2	LYS 377
ChemSpider ID65921	Amyrin	C_30_H_50_O	426,7	-705.0	-6.5	-	-709.2	-7.1	SER 375	-2165.3	-7.2	SER 372
ZINC13377636	Turmerone	C_15_H_20_O	216.32	-779.8	-6.6	GLN 493	-776.7	-6.7	THR 430	-2243.7	-7.6	HSD 364
ZINC4081455	Lupeol	C_30_H_50_O	426.72	0.0	0.0	-	-693.8	-6.0	ARG 355	0.0	0.0	-

## 4. Discussion

In a pandemic, natural compounds become an important source for the discovery of new antiviral agents. Several previous reports indicated that curcumin has broad spectrum antiviral activities. Several efforts were achieved to overcome the problems associated with bioavailability of curcumin. During the current study, we developed an immunomodulatory herbal extract with potent antiviral activity against SARS-CoV-2 by fusion of water extracts from curcumin with piperine.

EGYVIR showed virucidal effects on SARS-CoV-2. These results suggest that the virucidal mechanism is probably due to direct and strong attraction of EGYVIR extract to the virus spike present on the surface of SARS-COV-2. Consequently, this prevents the virus from attaching to VERO-E6 cells [23–26]. Moreover, it is possible that turmeron, lupeol, and sitosterol present in EGYVIR extract effectively inhibit the surface protein of SARS-COV-2 virus upon direct interaction or links with virions of virus through its amino group moieties with glycoprotein fusion.

EGYVIR components docking data revealed that most of the components bind precisely and directly to the ACE2 binding motif in RBD of spike protein, which directly interferes with the binding of spike to ACE2 receptor, and to other sites which might affect the dynamic action of the protein or the stability of changing conformation and indirectly affect binding, note that Pentatriacontane was found to bind firmly and precisely with the lowest DELTA G (9.7 kcal/mol). This might be interfering with the dynamics of the trimeric spike structure movement and hence the binding to ACE2 receptors indirectly.

The P50 docking with each of the components of EGYVIR (except for lupeol) showed strong predicted binding in the site of DNA binding, which causes the downregulation of IL6 expression and therefore down regulates the cytokine storm as shown in the results and confirmed by many previous publications.

β-amyrin (oleanane-type pentacyclic triterpenoid) was previously reported to exert antiviral efficacies against an influenza A virus (IAV) and herpes simplex virus (HSV) [27]. In our analysis, the percentage of β-amyrin (9.4%) in EGYVIR extract likely supports its anti-viral activity, probably via attenuating the cellular oxidative mechanism. Also, the triterpenoids, referred to as phytosterols, exhibits a broad spectrum biological activities [28, 29]. For instance, lupeol is a pentacyclic triterpenoid with *in vivo* and *in vitro* anti-inflammatory, anti-angiogenic, anti-microbial, antiprotozoal, anti-proliferative and hypocholesterolemic efficacies [30]. Lupeol decreases the ROS level and recover the antioxidant enzyme activities in chemical-induced oxidative stress condition [31]. Although lupeol has weak antiviral activities, it was used as a lead drug to construct more effective compounds against IAV and HSV [32]. To our knowledge, lupeol antiviral activity against CoVs is not reported, so far. Nevertheless, lupeol (8.86%) in EGYVIR extract contributes probably to the overall anti-viral activity of the extract via direct or indirect mode of action(s).

β-sitosterol is an immunomodulatory phytosterol with reported anti-HIV activities (*in vivo and in vitro*) via stabilizing CD4+ T-lymphocyte counts, and a significant decreasing of interleukin-6 expression level [33]. Interestingly, β-sitosterol can attenuate *in vitro* chemical-induced hepatotoxicity [34] and cardiotoxicity by enhancing mitochondrial glutathione redox mechanism [35] that may help in decreasing the cytokine storm. In line with this, the inclusion of β-sitosterol (7.6%) in EGYVIR extract strongly supports independently or interdependently its *in vitro* antioxidative, hepatoprotective and anti-viral activities.

EGYVIR has low IC50 value of 0.57 μg/ml against SARS-CoV-2. Herein, we provide evidence(s) that supports a direct impact of EGYVIR extract on the IKK/NF-κB signaling pathway. The amount of EGYVIR required to suppress Huh7 cell growth has been correlated with its ability to block the nuclear translocation of p50 (subunit of NF-κB), to hinder IκB phosphorylation and its subsequent degradation. In line with Kasinski *et al*. findings [36], Curcumin alone, showed at least 10 times lower potency in all of the above-mentioned assays.

Although the NF-κB signaling pathway is involved as one of the curcumin targets, the direct inhibitory effect of curcumin on IKK protein catalytic activity has not been validated yet [37]. Our research identifies EGYVIR as a NF-κB p50 inhibitor derived from the natural product curcumin and piperine extract, for controlling the IL6 expression [15, 38–40]. Sitosterol, turmerone and lupeol were also previously shown to be controlling the NF-κB and IL-6 expression. Notably, curcumin was previously reported an inhibitor of several kinases as well, in particular protein kinase C, epidermal growth factor receptor tyrosine kinase, and mammalian target of rapamycin serine/threonine kinase [41, 42].

On the other hand, under normal physiological conditions, the cytokine levels are maintained by negative and positive feedback regulation of their expression in a steady-state [43]. A large amount of virus in the body will induce over-reacted innate and adaptive immune response, triggering extravagant cytokines release, and lymphocytes activation, namely cytokine storm [43]. The loss of negative regulation of the production of inflammatory cytokines leads in turn to drive a high positive feedback regulation, resulting in exponentially growing inflammation and multi-organ failure. The release of cytokine promotes increased vascular permeability; consequently, the leukocytes increasingly migrate to damaged tissues through margination, rolling, adhesion, transmigration, and chemotaxis [44]. There is clear evidence from coronavirus-infected patients of both high cytokine levels and pathological changes in the lung [45–47]. For instance, in plasma of COVID-19 patients, high concentrations of IL-2, IL-6, and IL-7 were observed [48, 49].

Numerous studies showed that curcumin and its analogs significantly inhibit the production and release of pro-inflammatory cytokines *in vitro* and *in vivo* [10, 50]. In line with this, Zhang and his colleagues observed that direct pulmonary delivery of solubilized curcumin dramatically downregulate pro-inflammatory cytokines *in vitro and in vivo* in mice with severe pneumonia [51].

**Fig 9:**
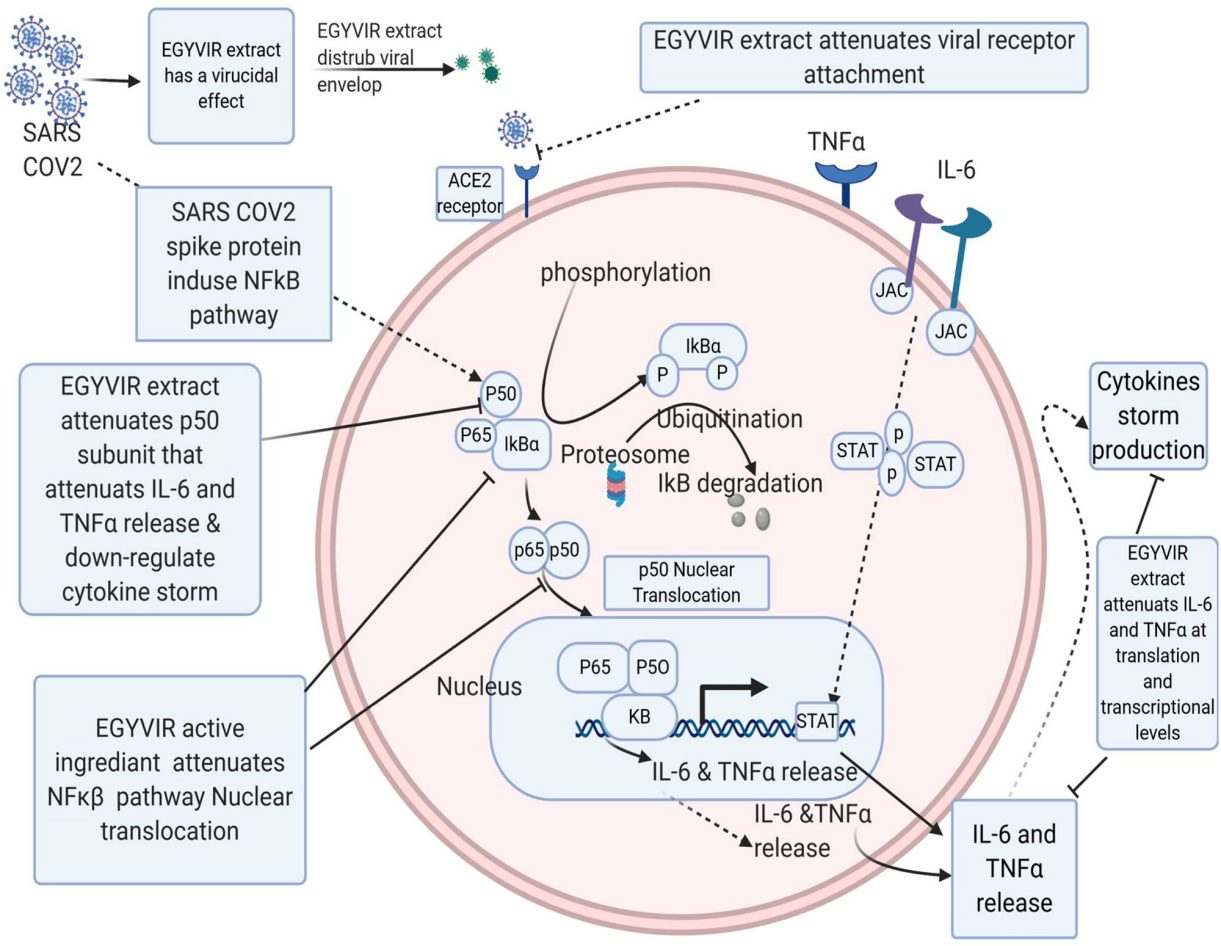
EGYVIR molecular mechanism as a virucidal and cytokine storm disturbance via NF-Kb pathway. EGYVIR works as a virucidal agent on SARS-COV-2 targeting the spike protein which prevent interaction with host cell receptor. Our docking studies showed that there are good bind affinities between EGYVIR extract active ingredients and RBD of human ACE2 receptor which disturbs the attachment of SARS-COV-2 virus with host, also our docking studies showed a good binding affinity between EGYVIR active ingredients and p50 subunit of NF-κB which attenuates NF-κB pathways. Our lab studies showed that EGYVIR inhibits the nuclear translocation of p50 and disturbs NF-κB pathway resulting in decrease of cytokine storm by down-regulating IL-6 and TNFα production.

The mechanism principal of EGYVIR extract has been explored as illustrated in Fig 9. Among inflammatory pathway during SARS-CoV2 infection, NF-kB plays an essential role in developing of cytokine storm. EGYVIR extract blocked this pathway by inhibition of nuclear translocation of NF-kβ p50 in line with Xu et al. findings, [52]. In line with our finding, it was reported that curcumin has ability to regulate NF-kB signaling through IKKb [53]. Additionally, consumption of curcumin reduced activity of IKKb in a study of patients with head and neck cancer and this was associated with a decrease in the expression of IL-8, TNF-α, and IFN-g [54]. Our docking studies showed that there are good bind affinities between EGYVIR extract active ingredients and RBD of human ACE2 receptor which disturbs the attachment of SARS-COV-2 virus with host, also our docking studies showed a good binding affinity between EGYVIR active ingredients and p50 subunit of NF-κB which attenuates NF-κB pathways. Our lab studies showed that EGYVIR inhibits the nuclear translocation of p50 and disturbs NF-κB pathway resulting in decrease of cytokine storm by down-regulating IL-6 and TNFα production. It has been documented that curcumin blocks NF-kB signaling upon infection with Influenza A virus (IAV) as a consequence of AMPK activation [55].

## 5. Conclusions

A curcumin-piperine infusion showed an immunomodulatory activity during *in vitro* infection of SARS-CoV-2. This extract also showed a potent virucidal effect. This is a potentially useful drug to respond to the COVID-19 pandemic after proper testing *in vivo* and in clinical trials.
